# O014: A new generation of hybrid biomaterials for antimicrobial medical devices

**DOI:** 10.1186/2047-2994-2-S1-O14

**Published:** 2013-06-20

**Authors:** N Theilgaard, A Svensson, A Olsen, P Thomsen, M Alm

**Affiliations:** 1 Danish Technological Institute, Denmark; 2 Biomodics ApS, Taastrup, Denmark

## Introduction

The present effective treatment for device related infections is large doses of systemically applied drugs. There is a high risk of recurring infections and biofilm formation for patients dependent on long term in-dwelling catheters and the extended dependency on antibiotics results in clinical drug resistance. We present a new tool for minimizing drug resistance by upgrading existing and future medical devices through a unique self-regenerating surface that prevents biofilm formation.

## Methods

An Interpenetrating Polymer Network (IPN) is composed of two or more networks which are at least partially interlaced on a molecular scale, but not covalently bonded to each other and cannot be separated unless chemical bonds are broken. In the present context, a unique approach for preparing silicone hydrogel IPNs is employed using supercritical carbon dioxide (scCO2) technology, which allows injection moulded and extruded silicone elastomers to be applied as substrates and uses scCO2 as an auxiliary solvent to impregnate the hydrophilic monomer into the silicone. The loading of an active substance is achieved by either incorporating the substance during the hydrogel synthesis process, or in a subsequent loading step, by swelling the material in a solvent containing the substance.

## Results

Release experiments of a hydrophilic dye from various IPNs show that it is possible to change the release characteristics by altering the hydrogel chemistry. An amount of 80% of the hydrophilic dye was released after 21 days by means of an IPN made of silicone and hydrophilic hydrogel. The IPN technology can further be used to alter mechanical properties of the device by making the substrate stiffer or softer, depending on hydrogels and process conditions. Interesting results have been obtained by preliminary tensile testing studies. µCT-scans show that the treatment is applied throughout the bulk of the material.

## Conclusion

A hybrid polymer with a storage facility and transport network has been produced showing very promising results regarding loading and release of active components from the surface. This system may be used for controlled, local and sustained delivery of drugs to combat catheter-related infections and avoidance of antibiotic resistance development.

## Disclosure of interest

None declared.

